# From Recommendation to Reality: The Follow‐Up Gap in Coeliac Disease

**DOI:** 10.1002/ueg2.70208

**Published:** 2026-03-24

**Authors:** Juha Taavela

**Affiliations:** ^1^ Department of Gastroenterology and Alimentary Tract Surgery Tampere University Hospital Tampere Finland; ^2^ Celiac Disease Research Center, Faculty of Medicine and Health Technology Tampere University Tampere Finland

Diagnosis of coeliac disease is in most cases straightforward. Serological testing for coeliac‐specific antibodies is performed, followed by duodenal biopsy when necessary [1]. Treatment is also relatively simple and consists of initiating a gluten‐free diet. But what happens thereafter? According to several studies, there is an obvious lack of long‐term follow‐up amongst patients with coeliac disease, with reported rates as low as 15% [2, 3, 4]. Many patients are lost to follow‐up, despite persistent symptoms in up to 30% of cases and ongoing duodenal mucosal damage in a considerable proportion of patients even after years of treatment [5, 6]. Furthermore, concerns remain regarding long‐term bone health and fracture risk, potential problems in reproductive outcomes, and overall nutritional status [7, 8, 9]. The new guidelines on coeliac disease management, follow‐up and complex disease courses therefore emphasise the importance of follow‐up to address these concerns [10].

Why and how should follow‐up in patients with coeliac disease be organized? One might assume that systematic follow‐up and counselling in coeliacs would have clear long‐term health benefits; however, there is a paucity of data demonstrating that follow‐up translates into measurable improvements in health outcomes [10]. Nevertheless, it appears logical to monitor these patients and provide appropriate counselling, although the optimal methods and intensity of follow‐up remain unclear. The primary aim of follow‐up is to identify patients with ongoing malabsorption or nutritional deficiencies, as well as those with severe disease manifestations such as refractory coeliac disease. These individuals are most likely to benefit from targeted interventions aimed at preventing complications. Consequently, the first follow‐up appointments are crucial for identifying patients who require closer attention. Based on the response to a gluten‐free diet and early follow‐up findings, a risk‐based and individualized approach appears appropriate thereafter, as emphasized in the guidelines [10].

However, an important question remains: how to engage both patients with coeliac disease and treating physicians in long‐term follow‐up [2, 3, 4]. The current level of follow‐up amongst patients with coeliac disease remains disturbingly low, and further research is needed to determine which follow‐up strategies provide meaningful health benefits while remaining feasible in clinical practise. As the population of patients with coeliac disease continues to grow, follow‐up care is likely to shift increasingly to primary care, with specialists managing mainly the more complex or refractory cases [11, 12]. Hence, improved implementation of follow‐up in routine clinical care is urgently needed, but the methodology needs further exploration and likely additional tailoring at the national level, as suggested by the authors of the new guidelines [10]. An additional emerging consideration is the potential introduction of pharmacological therapies for coeliac disease in the future [13]. If such treatments become available, they may influence follow‐up strategies, such as the need for a follow‐up biopsy before adjunctive drug treatment. However, until these therapies are approved, optimization of gluten‐free diet remains of utmost importance for coeliacs with drug treatments reserved only for those with refractory coeliac disease. In conclusion, suggested follow‐up schemes are only as effective as their implementation, underscoring the need for improved education and awareness amongst both patients and physicians. In addition, future research should seek to determine the most beneficial, but also feasible, follow‐up practices for improving the well‐being of patients with coeliac disease.

## Conflicts of Interest

J.T. is a Trainee Editor at the United European Gastroenterology Journal and has served as a consultant and/or lecturer for the Finnish Celiac Disease Society, Dr. Falk Pharma, Teva Pharmaceuticals, Anaptysbio, and PathAI.

## Data Availability

The author has nothing to report.
